# Two Coiled-Coil Proteins, WEB1 and PMI2, Suppress the Signaling Pathway of Chloroplast Accumulation Response that Is Mediated by Two Phototropin-Interacting Proteins, RPT2 and NCH1, in Seed Plants

**DOI:** 10.3390/ijms18071469

**Published:** 2017-07-08

**Authors:** Noriyuki Suetsugu, Masamitsu Wada

**Affiliations:** 1 Graduate School of Biostudies, Kyoto University, Kyoto 606-8502, Japan; 2 School of Science and Engineering, Tokyo Metropolitan University, Tokyo 192-0397, Japan; masamitsu.wada@gmail.com

**Keywords:** Arabidopsis, blue light, *Marchantia*, organelle movement, phototropin

## Abstract

Chloroplast movement is induced by blue light in a broad range of plant species. Weak light induces the chloroplast accumulation response and strong light induces the chloroplast avoidance response. Both responses are essential for efficient photosynthesis and are mediated by phototropin blue-light receptors. J-DOMAIN PROTEIN REQUIRED FOR CHLOROPLAST ACCUMULATION RESPONSE 1 (JAC1) and two coiled-coil domain proteins WEAK CHLOROPLAST MOVEMENT UNDER BLUE LIGHT 1 (WEB1) and PLASTID MOVEMENT IMPAIRED 2 (PMI2) are required for phototropin-mediated chloroplast movement. Genetic analysis suggests that JAC1 is essential for the accumulation response and WEB1/PMI2 inhibit the accumulation response through the suppression of JAC1 activity under the strong light. We recently identified two phototropin-interacting proteins, ROOT PHOTOTROPISM 2 (RPT2) and NPH3/RPT2-like (NRL) PROTEIN FOR CHLOROPLAST MOVEMENT 1 (NCH1) as the signaling components involved in chloroplast accumulation response. However, the relationship between RPT2/NCH1, JAC1 and WEB1/PMI2 remained to be determined. Here, we performed genetic analysis between RPT2/NCH1, JAC1, and WEB1/PMI2 to elucidate the signal transduction pathway.

## 1. Introduction

Phototropins (phot) are blue-light photoreceptor kinases that mediate phototropism, leaf flattening, stomatal opening, and chloroplast movement including low light-induced chloroplast accumulation response and strong light-induced chloroplast avoidance response (herein referred to as the accumulation and avoidance response, respectively). These responses contribute to optimal photosynthetic light utilization at the organ/tissue, cellular, and organelle level [1,2]. Most land plants have two or more phototropin genes and functional differences exist between them. There are two phototropins in *Arabidopsis thaliana*, phot1 and phot2. Phototropism, leaf flattening, stomatal opening, and the accumulation response are mediated by both phot1 and phot2; however, phot1 plays a greater role in these responses, especially at lower blue-light intensities [1,2]. In contrast, the avoidance response is mediated primarily by phot2 [1,2]. Clear functional divergence of phototropins in the accumulation and avoidance response is observed in the moss *Physcomitrella patens* and the fern *Adiantum capillus-veneris* [3,4]. However, in the liverwort *Marchantia polymorpha*, which is a basal land plant, a single phototropin mediates both the accumulation and avoidance responses [5]. Thus, phototropins can intrinsically mediate all phototropin-mediated responses and functional diversification of phototropins seems to have occurred during land plant evolution concomitant with phototropin gene duplication [6].

**Figure 1:**
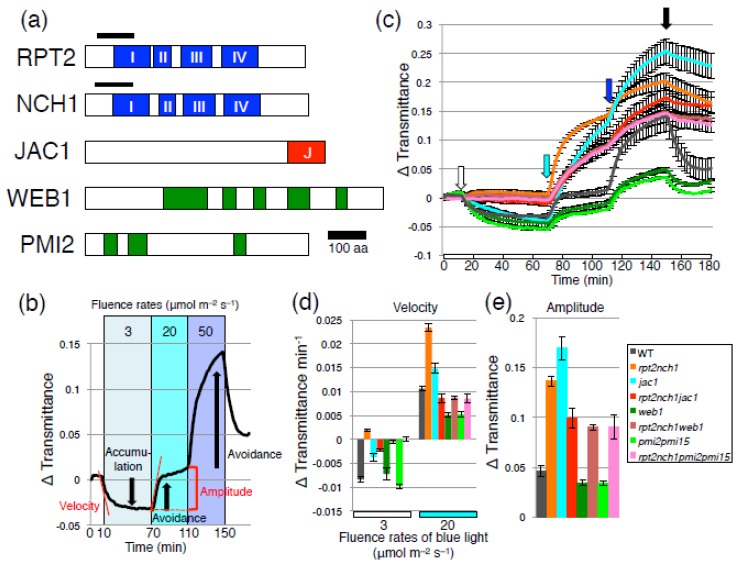
(**a**) Protein structure of ROOT PHOTOTROPISM 2 (RPT2), NRL PROTEIN FOR CHLOROPLAST MOVEMENT 1 (NCH1), J-DOMAIN PROTEIN REQUIRED FOR CHLOROPLAST ACCUMULATION RESPONSE 1 (JAC1), WEAK CHLOROPLAST MOVEMENT UNDER BLUE LIGHT 1 (WEB1), and PLASTID MOVEMENT IMPAIRED 2 (PMI2). **Blue** boxes indicate the four conserved regions of NPH3/RPT2-like (NRL) proteins. The position of the BTB/POZ domain is indicated by a **black** bar. **Red** box is a J-domain. **Green** boxes indicate the coiled-coil domains; (**b**) Measurement of light-induced changes in leaf transmittance as a result of chloroplast photorelocation movements. The depicted trace represents typical data collected for wild type under the various light irradiation conditions (indicated by color boxes). There is a decrease in leaf transmittance in response to 3 μmol m^−2^ s^−1^ of blue light, indicating that the accumulation response is induced (**downward arrow**). Conversely, there is an increase in leaf transmittance in response to 20 and 50 μmol m^−2^ s^−1^ of blue light, indicating that the avoidance response is induced (**upward arrows**). **Red lines** mark the initial linear fragments of leaf transmittance rate change during the first 2–6 min of the irradiation period, indicating the velocity. A **red parenthesis** marks the difference between the transmittance level observed following 60 min of 3 μmol m^−2^ s^−1^ blue-light irradiation and the transmittance level observed a following further 40 min of 20 μmol m^−2^ s^−1^ blue-light irradiation, indicating the amplitude of the avoidance response caused by 20 μmol m^−2^ s^−1^ blue-light irradiation; (**c**–**e**) Distinct chloroplast movements observed between *rpt2nch1* and *jac1*; (**c**) Light-induced changes in leaf transmittance of the indicated lines were measured using a custom-made plate reader system [14]. The samples were sequentially irradiated with 3, 20 and 50 μmol m^−2^ s^−1^ of continuous blue light. The beginning of each irradiation period is indicated by **white**, **cyan** and **blue** arrows, respectively. The light was extinguished after 150 min (**black arrow**); (**d**) The velocity of light-induced transmittance changes. (**e**) The amplitude of the avoidance response caused by 20 μmol m^−2^ s^−1^ blue-light irradiation. Data for wild type, *rpt2nch1*, *jac1* and *rpt2nch1jac1* from Suetsugu et al. (2016) [12] were used for comparison, because data for *web1*, *rpt2nch1web1*, *pmi2pmi15* and *rpt2nch1pmi2pmi15* were acquired in the same experiments using the same plate. Data are presented as means of three independent experiments and the error bars indicate standard errors. WT, wild type.

By regulating various signaling components, such as phototropin-interacting proteins, phototropins can regulate multiple, diverse responses. For example, BLUE LIGHT SIGNALING1 kinase is a direct phototropin substrate and specifically mediates stomatal opening [7]. In addition, the two phototropin-interacting bric á brac, tramtrack and broad complex/pox virus and zinc finger (BTB/POZ) domain proteins NONPHOTOTROPIC HYPOCOTYL 3 (NPH3) and ROOT PHOTOTROPISM 2 (RPT2), which belong to the NPH3/RPT2-like (NRL) protein family, mediate phototropism and leaf flattening [8,9,10,11]. Recently, we identified a phototropin-interacting NRL protein, NRL PROTEIN FOR CHLOROPLAST MOVEMENT 1 (NCH1), and found that NCH1 specifically mediates the accumulation response [12]. NCH1 is highly similar to RPT2 than NPH3 and contains four conserved regions including a BTB/POZ domain (Figure 1a). Furthermore, functional redundancy was found between NCH1 and RPT2 for the accumulation response, but not the avoidance response [12]. These results indicate that phototropism, leaf flattening, and the accumulation response are dependent on these NRL proteins, while stomatal opening and the avoidance response are independent of these NRL proteins. In *M. polymorpha*, the RPT2/NCH1 ortholog MpNCH1 specifically mediates the accumulation response, indicating that phototropin-regulated chloroplast movement is conserved in land plants [12].

Similar to NPH3 and RPT2, NCH1 is localized on the plasma membrane and interacts with phototropins [12], but the downstream function of NCH1 as well as other NRL proteins remained to be determined. Here, we performed genetic analysis of *RPT2* and *NCH1* using triple or quadruple mutant plants between *rpt2nch1* and other mutants that were implicated in the signal transduction of chloroplast movement.

## 2. Results and Discussion

**Figure 2:**
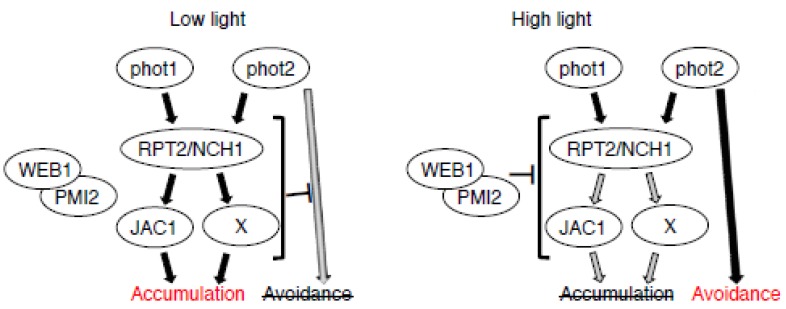
Working model of chloroplast photorelocation movements. The photoreceptors phot1 and phot2 mediate the accumulation response under a low light condition through RPT2 and NCH1. RPT2 and NCH1 might regulate both JAC1-dependent and -independent (X) pathways. The signaling pathway by RPT2/NCH1 and JAC1 suppresses that of the avoidance response under a low light condition. Under the high light condition, the WEB1/PMI2 complex suppresses the signaling pathway for the accumulation response that is regulated by RPT2/NCH1 and JAC1 through an unknown mechanism, resulting in the efficient induction of the avoidance response mediated by phot2. **Gray** arrows indicate the suppressed signaling pathways. **Black** arrows indicate the activated signaling pathways.

*J-domain protein required for chloroplast accumulation response* 1 (*jac1*) and *rpt2nch1* plants are both defective in the accumulation response [12,13]. JAC1 protein is a C-terminal J-domain protein similar to clathrin uncoating factor auxilin (Figure 1a) [13]. To analyze chloroplast movement in *jac1* and *rpt2nch1* in detail, we performed analysis of the light-induced changes in leaf transmittance, reflective of light-induced chloroplast movements (Figure 1b) [14]. In response to 3 μmol m^−2^ s^−1^ of blue light, which induces the accumulation response in wild type, a clear avoidance response is induced in *rpt2nch1* but not in *jac1* (Figure 1c,d) [12]. Therefore, RPT2/NCH1 could suppress the induction of the avoidance response to facilitate efficient induction of the accumulation response under low light conditions (Figure 2). Compared to wild type and *jac1*, a faster avoidance response was induced by 20 µmol m^−2^ s^−1^ of blue light in *rpt2nch1* (Figure 1c,d; one-way ANOVA followed by Tukey–Kramer multiple comparison post hoc test, *p* < 0.01 for wild type or *jac1* vs. *rpt2nch1*), although similar transmittance changes (defined as “amplitude” in Figure 1e) were observed for both *rpt2nch1* and *jac1* following 40 min of 20 µmol m^−2^ s^−1^ blue-light irradiation (Figure 1c,e; one-way ANOVA followed by Tukey–Kramer multiple comparison post hoc test, *p* > 0.05 for *jac1* vs. *rpt2nch1*). Following subsequent application of 50 µmol m^−2^ s^−1^ blue light, only a slight additional avoidance response was observed in *rpt2nch1* which contrasted with the stronger avoidance response induced in wild type (Figure 1c) [12]. This result could be attributed to prior movement of the majority of chloroplasts to the side walls in *rpt2nch1* during the former irradiation period. Interestingly, avoidance responses of similar magnitudes were induced in *jac1* in response to both 20 and 50 µmol m^−2^ s^−1^ of blue light and a decreased rate of avoidance response induction was not observed during strong light irradiation (Figure 1c). The changes observed in leaf transmittance for *rpt2nch1jac1* were intermediate between those observed for *rpt2nch1* and *jac1*.

Previously, we showed that *JAC1* mutation suppresses the defective avoidance response in *weak chloroplast movement under blue light 1* (*web1*) and *plastid movement impaired 2* (*pmi2*) [15]. WEB1 and PMI2 are related coiled-coil domain proteins that interact with each other (Figure 1a) [15]. Although the low light-induced accumulation response was normal in *web1* and *pmi2pmi15*, both mutant plants exhibited attenuated avoidance response under the strong light conditions (Figure 1c to e) [15]. The *jac1web1* and *jac1pmi2pmi15* showed nearly the same phenotypes as *jac1* single mutants [15]. Importantly, the weak avoidance response phenotype observed in *web1* and *pmi2pmi15* was completely suppressed in *jac1web1* and *jac1pmi2pmi15*, respectively [15]. Therefore, we hypothesized that WEB1 and PMI2 suppress JAC1 function under strong light, preventing the induction of the JAC1-dependent accumulation response and leading to efficient induction of the avoidance response (Figure 2). The weak avoidance response phenotype observed in *web1* and *pmi2pmi15* was absent in *rpt2nch1web1* and *rpt2nch1pmi2pmi15*, similar to *jac1web1* and *jac1pmi2pmi15* (Figure 1c–e). Mutation of *JAC1* suppressed *web1* and *pmi2pmi15* phenotypes, because *jac1web1* and *jac1pmi2pmi15* phenotypes are indistinguishable from *jac1* [15]. Although mutation of *RPT2* and *NCH1* largely suppressed the weak avoidance response phenotypes observed in *web1* and *pmi2pmi15*, the velocity and amplitude of the avoidance response in these mutants did not match those in *rpt2nch1* (Figure 1c–e; one-way ANOVA followed by Tukey–Kramer multiple comparison post hoc test, *p* < 0.01 for *rpt2nch1web1* or *rpt2nch1pmi2pmi15* vs. *rpt2nch1* in velocity and *p* < 0.05 for *rpt2nch1web1* or *rpt2nch1pmi2pmi15* vs. *rpt2nch1* in amplitude). The phenotypes of *rpt2nch1web1* and *rpt2nch1pmi2pmi15* were very similar to *rpt2nch1jac1* in that no detectable chloroplast movement was observed under 3 μmol m^−2^ s^−1^ of blue light and their avoidance response phenotypes were similar to *jac1* (Figure 1c,d; one-way ANOVA followed by Tukey–Kramer multiple comparison post hoc test, *p* > 0.05 for *rpt2nch1web1* or *rpt2nch1pmi2pmi15* vs. *rpt2nch1jac1*). Collectively, our results indicate that RPT2 and NCH1 are essential for the accumulation response and regulate JAC1-dependent and -independent pathways and that WEB1/PMI2 represses the signaling pathway for the accumulation response under the strong light conditions (Figure 2). However, how WEB1 and PMI2 suppress the accumulation response pathway remained to be determined. Interaction of WEB1 and/or PMI2 with JAC1 has never been detected [15]. At the least, the amounts of phototropins were normal in *web1* and *pmi2pmi15* mutant plants [15]. Further analysis of the relationship between WEB1/PMI2, RPT2/NCH1 and JAC1 is required.

RPT2 and NCH1 are localized on the plasma membrane and interact with phototropins, indicating that RPT2 and NCH1 are the initial downstream signaling components involved in the phototropin-mediated accumulation response (Figure 2) [12]. Notably, RPT2 and NCH1 are conserved in land plants, but JAC1, WEB1 and PMI2 orthologs are found only in seed plants [16]. Thus, to maximize light utilization through chloroplast movement, land plants have evolved a sophisticated mechanism of controlling chloroplast movement by increasing the molecular components involved in blue-light signaling.

## 3. Materials and Methods

### 3.1. Arabidopsis Lines and the Growth Condition

The wild-type and mutant lines are a Columbia *gl1* background. Seeds were sown on 0.8% agar medium containing 1/3 strength Murashige & Skoog salt and 1% sucrose, and grown under white light at ca. ~100 µmol m^−2^ s^−1^ (16 h)/dark (8 h) cycle at 23 °C in an incubator. *rpt2-4nch1-1* [12], *jac1-1* [13], *web1-2* [15] and *pmi2-2pmi15-1* [15] were described previously. For *PMI2* mutant plants, *pmi2pmi15* was used, because PMI15 is closely related to PMI2 and *pmi15* exhibits a very weak defect in chloroplast movement [17]. *rpt2-4*, *nch1-1*, *pmi2-2* and *pmi15-1* are T-DNA knockout lines [12,17]. *jac1-1* carries a missense mutation [13] and *web1-2* carries a deletion of one nucleotide [15]. Western blot analysis showed that JAC1 and WEB1 proteins were not detected in *jac1-1* and *web1-2*, respectively [13,15]. Double, triple and quadruple mutants were generated by genetic crossing.

### 3.2. Analyses of Chloroplast Photorelocation Movements

Chloroplast photorelocation movements were analyzed by the measurement of light-induced changes in leaf transmittance as described previously [14]. Third leaves that were detached from 16-day-old seedlings were placed on 1% (*w/v*) gellan gum in a 96-well plate and then dark-adapted at least for 1 h before transmittance measurement.

### 3.3. Statistical Analysis

Statistical analyses were performed by one-way ANOVA followed by Tukey–Kramer multiple comparison post hoc test.
